# Efficacy of Ultrasound for Localized Fat Treatment on Clinical and Psychological Outcomes: A Randomized, Single-Blind, Placebo-Controlled Clinical Trial

**DOI:** 10.1093/asjof/ojaa012

**Published:** 2020-03-24

**Authors:** Jaqueline Santos Silva Lopes, Sinara Pereira dos Santos, Lívia Maria Borges de Almeida, Ariadne Pereira Kayser, Elcilene Franciele Oliveira Reis, Ketelly Alves de Oliveira, Mirella Carina do Amaral Queiroz, Luaneia Pereira da Silva, Ana Beatriz Ferreira Marques, Bethânia Monteiro da Silva Borges, Aline Castilho de Almeida

**Affiliations:** 1 Department of Physiotherapy, University Center of the Araguaia Valley (UNIVAR), Barra do Garças, Brazil; 2 Department of Aesthetics, Center University of the Araguaia Valley (UNIVAR), Barra do Garças, Brazil; 3 Department of Physiotherapy, Federal University of São Carlos (UFSCAR), São Carlos, SP, Brazil

## Abstract

**Background:**

The use of ultrasound for localized fat treatment on possible psychological influences is little explored to date. Therefore, it is relevant to elaborate studies that include a placebo group in order to measure the real effects of the exclusive application of ultrasound.

**Objectives:**

To verify the influence of ultrasound application for localized fat treatment on clinical, functional, and psychological outcomes.

**Methods:**

Thirty female participants who were candidates for localized abdominal fat treatment were included and randomly divided into three groups: control (CG, *n* = 10), experimental (EG, *n* = 10), and placebo (PG, *n* = 10). The CG did not receive any intervention. The EG received 10 ultrasound sessions for 20 minutes. For the PG, ultrasound was also applied for 20 minutes, but with zero intensities. Anthropometric assessment, cardiovascular parameters, circumference measurements, photography, endurance test, and subjective questionnaires were performed before and after the treatment protocols.

**Results:**

The EG photographs show an improvement of 60% in the visual appearance. Regarding the other analyzed outcomes, no statistically significant differences were found between moments and groups (*P* > 0.05).

**Conclusions:**

Pretreatment and posttreatment photographs analysis demonstrate visual improvement in the appearance of abdominal localized fat in the EG. However, ultrasound application, when compared with CG and PG, is not a superior method for improving clinical, functional, and psychological parameters.

**Level of Evidence: 2:**

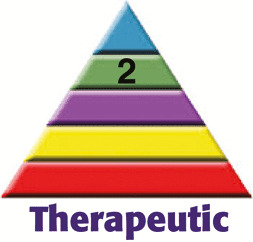

The accumulation of body fat in specific regions of the female body characterizes a condition related to negative aspects in health psychological variables.^1^ Its etiology includes sedentary lifestyle, heredity, inadequate eating habits, and the presence of metabolic diseases. This condition is also responsible for low self-esteem, personal satisfaction, quality of life, and shame, besides being a risk factor for the onset of cardiovascular and musculoskeletal diseases. Thus, noninvasive treatments for localized fat reduction characterize indispensable management in the practice of body therapy and aesthetic physiotherapy.^2^

In this respect, the accumulation of adipose tissue, in addition to causing the mentioned aesthetic dissatisfactions, also compromises the organism’s functionality.^3^ Thus, the functional outcomes investigation resulting from the ultrasound application is important, considering the importance of maintaining resistance and strength conditioning in the abdominal region due to its influence on other body segments, being important for the breath control, balance, posture and provides the basis that makes compensatory mechanisms reduced in adjacent body segments.^3^ In addition, clinical measurements such as the waist/hip ratio (WHR) are used as predictors of risk factors for the onset of cardiovascular disease^4^ and can be used as an evaluation parameter during clinical treatments.

In this sense, the presented dysfunction treatment includes several behaviors, on which they must necessarily be associated with healthy habits that include a balanced diet and continuous physical activity practice. With regard to aesthetic care, the literature currently presents different strategies for the treatment of localized fat, including cryolipolysis,^5^ electrolipophoresis,^6^ radiofrequency,^7^ electrostimulation,^8^ ultrasound,^9^ and manual therapy resources.^10^

Among the mentioned methods, high-frequency ultrasound stands out as a focal tool, which uses sound waves in a “localized” mode without energy dispersion in the treated region, respectively, with the purpose of triggering lipolysis in adipocytes, with subsequent fat tissue reduction. Thus, once the adipocyte membrane is broken, triglycerides are released into the intercellular space, where free fatty acids are oxidized in the tissues that require energy or transported to the liver, where they are metabolized.^9^

In this scenario, studies investigate the clinical efficacy of ultrasound specifically for localized fat treatment,^2,11^ demonstrating positive results when used alone or in combination with another technique. However, the protocols used are inconclusive, poorly standardized, and have no validation report or scientific evidence specifically regarding psychological and functional outcomes.^2,9,11^ This fact is an important limitation on the reliability of the use of this method in clinical practice, and it seems appropriate to elaborate studies that go beyond this barrier, providing accurate evidence.

Also, in response to advances in technological and scientific innovations in the search for desired outcomes, it appears that the number of noninvasive treatments in the aesthetics area has been the majority choice, due to the security offered in these types of procedures and lowest financial cost.^12^

From the above, there are good results in the literature on the use of ultrasound in the localized fat treatment. However, the gaps presented make it evident the need for studies that investigate the possible psychological and functional influences of this method and, therefore, it is believed that studies involving a placebo group are pertinent to measure the real effects of the exclusive application of this technique. Thus, the aim of this study was to verify the influence of ultrasound application on localized abdominal fat treatment on clinical, functional, and psychological outcomes.

## METHODS

### Participants

The recruitment of participants was conducted using local advertisements and social media. After verification of the inclusion criteria, in total 30 females, sedentary, aged between 18 and 30 years old, apparently healthy participants, were included. To define the sample size, a priori knowledge was used, based on the findings of Moreno et al.^13^ The chosen variable referred to body circumference values. For this, a 2-tailed hypothesis test was used with a significance level of 5% and 80% of power and possible sample loss of 15%. The stipulated sample size would correspond to 12 participants per group.

Additional inclusion criteria included body mass index (BMI) ≤26.0 kg/m^2^, adipose tissue thickness 2.6 cm or more in the treatment area, preserved elasticity, and local tissue integrity. In addition, participants had to agree not to change their daily routines during the study.

**Figure 1.**
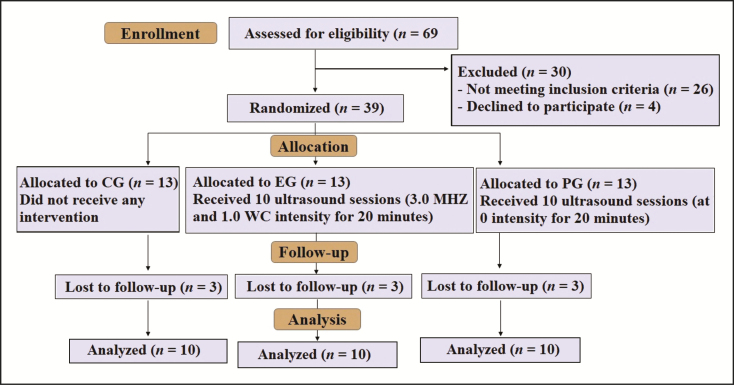
Flowchart of the study.

After follow-up losses, 30 participants (10 per group) had their data analyzed. All follow-up losses were due to lack of time to attend the scheduled assessments or intervention to the detriment of participants’ personal commitments. Participant recruitment and reasons for loss to follow up are shown in Figure 1.

Exclusion criteria were pregnancy; breastfeeding; history of liposuction; lipolysis by injection therapy; abdominoplasty or surgery in the treatment region; weight reduction medication; recent surgery in the last 12 months; implantable fixture; neurosurgical deviation; hernia, sensory loss, or dysesthesia in the treatment region; cancer; circulation problems; or chronic systemic diseases, such as diabetes or metabolic syndrome.

### Ethical Aspects and Study Registration

The study was approved by the Research in Human Ethics Committee of the Federal University of Mato Grosso, Araguaia campus, under the number of registry: 3,356,517. This study was also registered in the Clinical Trials (clinicaltrials.gov) to increase the visibility and transparency of procedures performed for the entire scientific community (Registration No. NCT04043182).

Participants were informed about the procedures prior to participation in the study; after agreeing to participate, they gave their informed consent. This study was conducted according to Resolution 466/12 norms of the National Health Council on research involving humans, ensuring their privacy rights.

### Study Design

This study is a randomized, single-blind, placebo-controlled trial. All procedures and data collection were conducted at the laboratories of the University Center of Vale do Araguaia (UNIVAR), from February to May of 2019, under standard environmental conditions (temperature: 23 ± 1°C; relative humidity: 84%).

An independent researcher not involved in the study procedures generated the participants’ randomization and allocation in the groups. Participants were randomized through a sealed envelope into three groups: CG (control group, *n* = 10), EG (experimental group, *n* = 10), and PG (placebo group, *n* = 10). In the EG, 10 sessions with Ultrasound (Skinner brand, São Paulo) were performed on the lower abdomen region for 20 minutes. In the PG, the participants received an identical GE application, however, with zero intensities, and thus no ultrasonic waves were propagated in the body tissue. The CG did not receive any intervention and only made 2 laboratory visits for evaluations.

Participants, researcher, and evaluator were blinded to the hypotheses and group allocation, as well as the analyzed outcomes. Thus, the control group was not aware of the characteristics of the other analyzed groups.

Prior to each measurement, participants were instructed to fast for at least 4 hours and wearing appropriate light clothing for evaluation. In order to maintain standardization of the procedures performed, each evaluator remained fixed in the same function throughout the study.

## MEASUREMENTS

**Figure 2.**
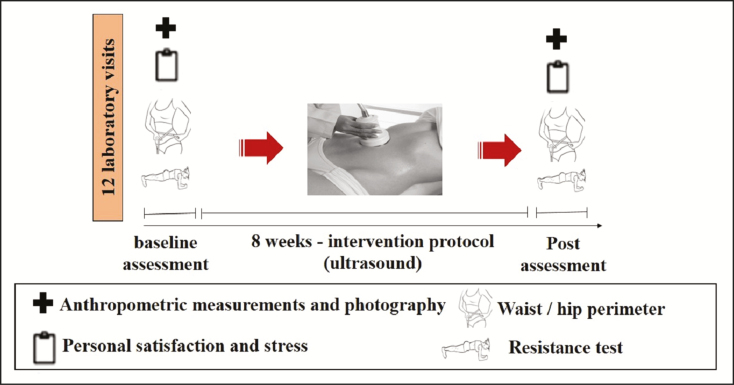
Study design.

Prior to the beginning of the treatment protocol (day 0), anthropometric measurements, waist and hip circumference, functional resistance, cardiovascular parameters, and subjective assessment of stress and personal satisfaction with the body were measured for each participant. The photograph was taken with a fixed focal length and under constant illumination. The images were evaluated by a double-blind method by 2 physiotherapists with expertise in the aesthetics area, who judged changes in fat located in the abdomen in the pictures. After protocol completion, all described measures were repeated. The general study design is shown in Figure 2.

The primary outcome was fat reduction in the treated region, based on the visual assessment by 2 blind investigators of randomized photographs in all groups of pretreatment vs posttreatment (after the 10th session), where the assessor chose which photograph would correspond, respectively, to the pretreatment and posttreatment moments.

### Procedures

#### Experimental Protocol

For the experimental protocol, a 3-Mhz high power Large Surface Ultrasound was used with 8 crystals, totaling 60 W of power, and cavitation in 950 Khz totaling 120 W of power (Skinner—São Paulo, Brazil).

The treatment protocol for the experimental groups (EG and PG) consisted of 10 sessions of ultrasound for 20 minutes. In the EG, the energy level corresponding to the intensity of 1.0 Wcm^2^ and frequency of 3 MHZ was used, in continuous mode, applied on the lower abdomen region, in a quadrant previously delimited. The flanks (lateral of the abdomen) were not treated. The described protocol parameters were used as suggested by Machado et al.^14^ In the PG, the same protocol was used, however, with zero intensities. The sessions were conducted twice a week, respecting the minimum interval of 48 hours between sessions, always in the morning, which totaled 8 weeks of follow-up.^15^

During the study, all participants, including the CG, were instructed to maintain balanced eating habits and not to perform additional physical activities or any kind of sports practice. Thus, this recommendation was reinforced weekly through reminders sent by text messages.

#### Anthropometric Measurements

A scale (Tanita BC-554, Ironman—Arlington Heights, IL, USA) and a stadiometer (Sanny, American Medical Brazil, Sao Paulo, Brazil) were used to assess anthropometric measures of the participants. BMI was calculated by dividing weight by the value of squared height [kg: (m^2^)*1].^16^

#### Personal Satisfaction and Stress

Personal satisfaction with the body and stress about the applied technique were measured using a questionnaire, which aimed to investigate such variables subjectively. Thus, the participants were instructed to draw a line on a visual analog scale of 10 cm between two extremes, with 0 being the “minimum possible” and 10 indicating “the most possible” for each classification.^12^

#### Comfort Level of the Technique and Satisfaction With the Treatment Performed

After the protocol application, the participants were asked about the comfort level of the applied technique and were asked to choose one of the following: intolerable, tolerable, comfortable, and very comfortable. Regarding satisfaction with the treatment, participants were encouraged to choose one of the following: disappointed, neutral, satisfied, or extremely satisfied.^17^

#### Functional Performance

For functional performance evaluation, the plank test was performed. The participant was asked to maintain the upper body supported off the ground by the elbows and forearms, and the legs straight with the weight taken by the toes. The hip was should be lifted off the floor creating a straight line from head to toe. As soon as the participant was in the correct position, the stopwatch was started. The participant was instructed to maintain head facing toward the ground and not looking forwards. The test was over when the participant was unable to hold the back straight and the hip is lowered. Time was measured in seconds, and better functional performance was related to longer test time. It is a previously validated test.^18^

#### Clinical Parameters

Blood pressure (BP) was measured on the nondominant arm after 5 minutes of rest in the sitting position. Measurements were performed using the auscultatory method with a sphygmomanometer and stethoscope, properly calibrated. Heart rate (HR) was also checked by a portable MONTSERRAT device under rest in the sitting position.

To measure values corresponding to the waist/hip ratio (WHR), a tape measure was used, which collected measurements in centimeters (cm). Thus, waist circumference was measured at the level of the umbilicus. Hip circumference was taken around the widest portion of the buttocks.^19^ For standardization purposes, such measures were always performed early in the morning and before the evaluations, the participants were instructed to eat light meals. In addition, the measurement was made at the same phase of the breathing cycle. The participants’ menstrual cycle phase was not controlled for logistical reasons.

### Statistical Analysis

Statistical analysis was performed using the SPSS 2.0 software package. Descriptive analysis was presented as mean and standard deviation values. Scores regarding the comfort level of the technique were analyzed descriptively (*n* [%]). Comparisons of the variables analyzed between the pretreatment and posttreatment period were calculated by the mean of the differences in percentage. Statistical significance between groups and moments was calculated using ANOVA. Effect size (ES) was calculated for post-intervention between groups. Cohen^20^ reports the following intervals for ES: 0.1 to 0.3: small effect; 0.3 to 0.5: moderate effect; 0.5 and higher: strong effect. Significant differences will be established with *P* <0.05 (5%).

## RESULTS

During the data collection, no type of discomfort, pain, or sensitivity alteration was recorded through the participants’ inspection and report at the proposed method application region.

The anthropometric characteristics of the participants are presented in Table 1. No significant differences were observed between groups (*P* > 0.05) for age, body mass, height, and BMI, which confirm the homogeneity of the sample.

**Table 1.** Participant’s Anthropometric Characteristics (Mean ± SD)

	CG (*n* = 10)	EG (*n* = 10)	PG (*n* = 10)	*P*-value
Age (years)	23 ± 3.1	22.6 ± 3.0	24.1 ± 3.5	0.632
Weight (kg)	65.4 ± 9.2	62.4 ± 6.2	66.7 ± 4.9	0.140
Height (m)	1.65 ± 0.1	1.60 ± 0.1	1.63 ± 0.1	0.061
BMI (kg.m^2^)	24.1 ± 3.0	23.9 ±2.1	24.3 ± 1.8	0.922

BMI, body mass index; CG, control group; EG, experimental group; PG, placebo group; *n*, number of participants; SD, standard deviation; m = meters; kg = kilograms; kg.m^2^ = kilograms per square meter.

**Figure 3.**
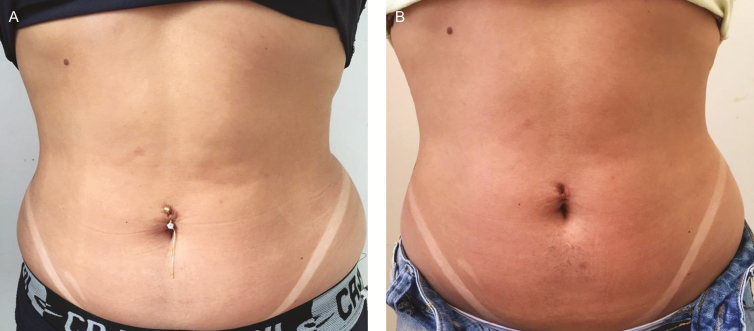
(A) A 26-year-old female participant with an initial BMI of 25.79. (B) Post 10 sessions of ultrasound treatment application after 8 weeks.

**Figure 4.**
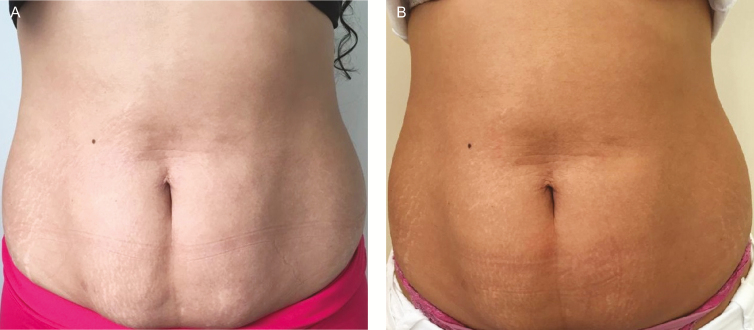
(A) A 25-year-old female participant with an initial BMI of 25.9. (B) Post 10 sessions of ultrasound treatment application after 8 weeks.

For the primary outcome, photographs were correctly identified by blinded researchers in 60% (6/10) of the evaluations in the EG, 90% (9/10) in the CG, and 30% (3/10) in the PG. An example of correct photography identification between moments in the experimental group is shown in Figures 3 and 4. The clothing of the participants in the evaluation sessions was not standardized and, therefore, they are presented with different clothing. Despite this, a control was implemented to guarantee the quality of the image, together with a professional in the area. In addition, the interval between images (8 weeks) was equivalent to the total treatment time. Written consent was acquired from each participant. 

The results of BP, HR, WHR, functional performance, stress, and personal satisfaction did not show a statistically significant difference between groups and moments (*P* > 0.05). Despite this, were observed highest percentage variations for all investigated variables in PG. All respective values are shown in Table 2.

**Table 2.** Clinical and Functional Performance Parameters, Personal Satisfaction, and Stress Pre- and Post-Intervention

Variables	CG (*n* = 10)		EG (*n* = 10)		PG (*n* = 10)	
	Mean ± SD	Δ%	Mean ± SD	Δ%	Mean ± SD	Δ%
SBP (mmhg)						
Pre	103.2 ± 7.5	7.5	103.7 ± 5.1	3.75	101.1 ± 6.0	15.56
Post	111.5 ± 7.5	7.5	107.5 ± 11.6	3.75	116.6 ± 8.6	15.56
DBP (mmhg)						
Pre	72.3 ± 8	1.4	68.7 ± 9.9	0	65 ± 6.1	10.6
Post	71.4 ± 8	1.4	68.7 ± 8.3	0	75.5 ± 5.5	10.6
HR (bpm)						
Pre	82.7 ± 13.6	−2.62	90.7 ± 12.8	−7.62	87.2 ± 14.3	−9.0
Post	80.1 ± 15.3	−2.62	83.1 ± 11.8	−7.62	78.2 ± 17.0	−9.0
WHR (cm)						
Pre	0.80 ± 0.05	0.02	0.77 ± 0.03	0.01	0.77 ± 0.10	−0.03
Post	0.82 ± 0.06	0.02	0.78 ± 0.03	0.01	0.78 ± 0.05	−0.03
Functional performance(s)						
Pre	42.1 ± 25.7	−5.37	39.5 ± 18.4	0.62	32.7 ± 12.5	2.44
Post	36.7 ± 19.7	−5.37	40.1 ± 13.7	0.62	35.3 ± 13.3	2.44
Stress (0–10)						
Pre	6.8 ± 2.1	1.37	7.3 ± 2.3	0.56	7.7 ± 1.2	0.11
Post	8.2 ± 1.1	1.37	6.7 ± 1.5	0.56	7.8 ± 1.5	0.11
Personal satisfaction (0–10)						
Pre	6.6 ± 1.5	−1.0	5.5 ± 2.6	0.37	5.8 ± 3.0	0.83
Post	5.6 ± 2.1	−1.0	5.6 ± 1.1	0.37	6.6 ± 2.0	0.83

CG, control group; EG, experimental group; HR, heart rate; PG, placebo group; *n*, number of participants; SD, standard deviation; WHR, waist/hip ratio; Δ, change from baseline; mmhg, millimeters of mercury; bpm, beats per minute; cm, centimeters; s, seconds.

Table 3 shows the results of effect size for each variable between groups on posttreatment evaluations. It was observed strong effects for group comparisons between CG and EG for hip-waist ratio (0.8) and stress (1.1); between CG and PG for systolic blood pressure (SBP) (0.6), diastolic blood pressure (DBP) (0.6), and hip-waist ratio (0.7); between EG and PG for SBP (0.9), DBP (0.9), stress (0.7), and personal satisfaction (0.6). The remaining comparisons showed small and moderate effects between groups. 

**Table 3.** Between Groups Effect Size for Each Variable on Posttreatment

	CG vs EG	CG vs PG	EG vs PG
SBP (mmhg)	0.4 (−1.3 to 0.5)	0.6 (−0.3 to 1.5)	0.9 (−0.03 to 1.8)
DBP (mmhg)	0.3 (−1.2 to 0.5)	0.6 (−0.3 to 1.5)	0.9 (0.04 to 1.9)
HR (bpm)	0.2 (−0.6 to 1.1)	0.1 (−0.9 to 0.8)	0.3 (−1.2 to 0.5)
WHR (cm)	0.8 (−1.7 to 0.1)	0.7 (−1.6 to 0.2)	0.0 (−0.9 to 0.9)
Functional performance(s)	0.2 (−0.7 to 1.1)	0.1 (−0.9 to 0.8)	0.3 (−1.2 to 0.5)
Stress (0–10)	1.1 (−2.1 to −0.2)	0.3 (−1.2 to 0.6)	0.7 (−0.2 to 1.6)
Personal satisfaction	0.0 (−0.9 to 0.9)	0.5 (−0.4 to 1.4)	0.6 (−0.3 to 1.5)

CG, control group; EG, experimental group; HR, heart rate; PG, placebo group; WHR, waist/hip ratio; mmhg, millimeters of mercury; bpm, beats per minute; cm, centimeters; s, seconds.

Table 4 presents percentage values corresponding to the level of comfort and satisfaction regarding the treatment performed in the EG and PG groups. Most of the participants considered the technique comfortable, with neutral satisfaction regarding the treatment performed.

**Table 4.** Comfort Level and Satisfaction With Technique During Treatment (%)

	CG (*n* = 10)	EG (*n* = 10)	PG (*n* = 10)
Comfort level			
Intolerable	—	0	0
Tolerable	—	0	0
Very tolerable	—	30.0	10.0
Comfortable	—	70.0	90.0
Satisfaction with technique			
Disappointed	—	10.0	0.0
Neutral	—	70.0	40.0
Pleased	—	10.0	60.0
Extremely satisfied	—	10.0	0.0

CG, control group; EG, experimental group; PG, placebo group; *n*, number of participants.

## DISCUSSION

To our knowledge, this is the first study that investigates cardiovascular parameters, WHR, and functional performance in response to the ultrasound application. The main results show that the application of ultrasound in the lower abdomen region resulted in satisfactory outcomes in the photographs evaluated in the experimental group. However, there were no statistically significant changes in clinical, functional, and psychological parameters in the EG when compared with PG and CG. In addition, the participants regarding the observed results considered the technique with reports of neutral satisfaction.

Regarding the outcomes related to photography analysis, satisfactory results were found in 60% of the photographs evaluated in the EG. Such finding may be justified by the physiological mechanism of action triggered by the application of high frequency focused ultrasound. In this sense, studies state that sound waves cause apoptosis of adipose cells in response to the intense heat produced at the region.^9,21^ Inflammatory mechanisms then start in the injured cell where macrophages phagocyte and transport the lipids and cell debris from the injured adipocytes, which are metabolized in the liver and subsequently eliminated by the body. The set of responses described then reduces the fat contingent in the treated region.

Also, the similar results observed between EG and PG suggest a possible influence of psychological effect on the verified responses. In this scenario, it is observed in the literature that the placebo condition has become increasingly prominent, because its effects constitute a well-accepted phenomenon in medicine, even used as a therapeutic intervention.^22^ In this regard, studies have shown that the placebo effect influences sports performance^22^ with a potentiated effect in long-term periods^23^ and the presented findings also indicate a possible influence on aesthetic protocol results.

Some parameters deserve to be considered and scientifically analyzed in future studies, such as proposed treatment time and different intensity and frequency. In this regard, although time and protocol parameters have been proposed in the literature, specific populations may require prescribing individual parameters according to specific biological needs.

In this sense, Gold et al^24^ evaluated the effects of a 16-week ultrasound application on flank reduction in men and women. The outcomes demonstrated that all participants significantly reduced fat in the treated area as measured by ultrasound and caliper. In addition, participants expressed high satisfaction with the treatment outcomes. The present study findings were similar when verifying improvement in the experimental group photographs. In contrast, unlike this study that found high satisfaction, our study recorded predominantly neutral satisfaction. One hypothesis for this is based on the different treatment duration, as our protocol lasted 8 weeks, which may have influenced the participants’ results perception. 

Similarly, other authors^1,25^ observed that the combination of bipolar radiofrequency energy and ultrasound cavitation technology had a significant positive effect on the reduction of abdomen and waist circumferences and leptin when used twice a week for 5 weeks^1^ and after only 3 treatment sessions.^25^ Although the treatment used had a shorter duration than the one used in the present study, the experimental group received combined radiofrequency energy and ultrasound cavitation technology interventions. In addition, in Arabpour-Dahoue et al^1^ study, both groups were implemented with the low-calorie diet, which may have helped to promote better results. Moreover, the studies were limited on not providing a group treated with ultrasound alone as well as a control group who received no intervention.

Regarding the analyzed outcomes, although no statistically significant differences were found between moments and groups, the best responses for the WHR variables, functional assessment, stress, and personal satisfaction were observed in PG and the worst in CG. This fact reiterates the need for future research to investigate the real physiological effects triggered by the application of ultrasound in this population.

Considering that this is a pioneer investigation in the area, great difficulty was observed when making specific comparisons with other studies. In addition, the observed outcomes trigger questions and create gaps regarding the need for future investigations that justify and correlate such results with other variables.

Blood and body composition analyses are essential measures to complement the evidence presented in this study and discuss their real impact on body fat reduction. In association, we believe that the demonstrated findings constitute an initial parameter and intrinsic potential to complement specific clinical outcomes by enabling particular physiological associations triggered by the application of the analyzed method.

In the current study, Lopes et al presented strengths. Firstly, it was elaborated from high methodological quality using a single-blind placebo-controlled design. In addition, all items in the CONSORT checklist were followed.^26^ Moreover, the outcomes presented are unpublished and constitute peculiar potential under new perspectives and parameters related to the area.

The main limitation of this study was that long-term follow-up after the intervention was not performed. However, in the near future we intend to conduct a new study with longer protocol time, comparing its effects with the present study, using the same outcomes and circumstances, but with different time periods and application. Second, we believe that not having controlled the menstrual cycle phase for WHR measurements may characterize a limitation that has partly influenced the findings, as some women report complaints of bloating during the premenstrual phase, which may affect circumference measurement data. Thirdly, only the abdomen region was treated, and the flanks region treatment may eventually lead to more noticeable changes. Finally, the lack of validated psychological questionnaires also deserves to be highlighted, in order to accentuate the need for specific studies that focus on this issue.

It is pertinent that future studies address the analysis of biochemical markers, such as hormonal rate, lipids, and cytokines, as well as body composition that provide a pattern of observed physiological responses in order to measure possible metabolic differences related to the clinical, functional, and psychological parameters demonstrated in this study. In addition, systematic review and meta-analysis studies on the subject are necessary in order to propose standardization on the parameters of ultrasound application, supported by good levels of scientific evidence.^27^

## CONCLUSIONS

From the presented findings, it is possible to conclude that the ultrasound protocol application, when compared with the control and placebo conditions, does not constitute a superior method in improving clinical, functional, and psychological parameters. Nevertheless, satisfactory alteration in the photographs of the experimental group was evidenced.

Moreover, the resulting outcomes support relevance by presenting important elements related to unpublished responses resulting from the application of ultrasound and comparing to the placebo condition what characterizes competence for reflection among the clinical and scientific communities, and society in general regarding clinical applicability and use of this method for the treatment of localized abdominal fat in young women.
